# Determinants of Cognitive Performance in Children and Adolescents: A Populational Longitudinal Study

**DOI:** 10.3390/ijerph19158955

**Published:** 2022-07-23

**Authors:** Rodrigo Antunes Lima, Fernanda Cunha Soares, Mireille van Poppel, Saija Savinainen, Aino Mäntyselkä, Eero A. Haapala, Timo Lakka

**Affiliations:** 1 Research, Innovation and Teaching Unit, Parc Sanitari Sant Joan de Déu, CIBERSAM, 08830 Sant Boi de Llobregat, Spain; 2 Division of Orthodontics and Pediatric Dentistry, Department of Dental Medicine, Karolinska Institutet, 171 77 Stockholm, Sweden; fercsoares@gmail.com; 3 Institute of Sport Science, University of Graz, 8010 Graz, Austria; mireille.van-poppel@uni-graz.at; 4 Institute of Biomedicine, School of Medicine, University of Eastern Finland, 70211 Kuopio, Finland; saija.savinainen@uef.fi (S.S.); eero.a.haapala@jyu.fi (E.A.H.); timo.lakka@uef.fi (T.L.); 5 Department of Pediatrics, Kuopio University Hospital, 70211 Kuopio, Finland; aino.mantyselka@kuh.fi; 6 Faculty of Sport and Health Sciences, University of Jyväskylä, 40014 Jyväskylä, Finland; 7 Department of Clinical Physiology and Nuclear Medicine, School of Medicine, Kuopio University Hospital, University of Eastern Finland, 70211 Kuopio, Finland; 8 Kuopio Research Institute of Exercise Medicine, 70100 Kuopio, Finland

**Keywords:** children, adolescents, cognition, development, behaviour, lifestyle, health

## Abstract

We evaluated the determinants of cognitive performance in children and adolescents. This is a longitudinal study, secondary analysis of the Physical Activity and Nutrition in Children (PANIC) study. We assessed 502 children (51.6% girls) at middle childhood (range: 6.6 to 9.0 years), at late childhood, 437 children (51.0% girls, range: 8.8 to 11.2 years), and in 277 adolescents (54.5% girls, range: 15.0 to 17.4 years). Raven’s progressive matrices tests estimated the participants’ cognitive performance (outcome variable) at all time points. In total, we evaluated 29 factors from various dimensions (prenatal, neonatal, child fitness, lifestyle and anthropometrics). None of the neonatal and anthropometric parameters were associated with cognitive performance. Preeclampsia (prenatal) and listening to music, writing, arts and craft and watching TV (lifestyle) were negatively associated with cognitive performance. Shuttle run and box and block tests (fitness), and playing music, reading and time at the computer (lifestyle) were positive determinants of cognitive performance in children and adolescents. Fitness and lifestyle factors during childhood and adolescence diminished the importance of prenatal factors on cognitive performance and lifestyle factors were especially relevant in regard to cognitive performance. Reading was positively associated with cognitive performance, regardless of age and time dedicated, and should be promoted.

## 1. Introduction

Cognitive performance encompasses the ability of processing information, intelligence and reasoning, along with language and memory development [1]. Cognitive development is crucial for adequate self-perception in relation to the social environment and essential for interpersonal connection and interaction [2]. Therefore, cognitive development is pivotal for cultivating our learning and adaptative skills [3]. Ultimately, enhanced cognitive development in youth is related to higher success at school, better job opportunities and higher income, resulting in better quality of life [4,5].

To date, longitudinal studies have reported various factors being associated with cognitive performance in children [6,7] and adolescents [8,9]. In summary, cognitive performance in children and adolescents has been associated with prenatal and neonatal factors as well as factors related to their childhood and adolescence periods, such as lifestyle, anthropometrics and fitness factors [6,7,8,9]. However, there are a number of limitations in the state of the art.

First, only a few studies have long-term follow-ups monitoring children until adolescence [6,7,8,9,10,11,12,13,14]. Second, although prenatal and neonatal factors have been related to later cognitive performance in early childhood [10,11], it is not well defined whether and how those factors continue to impact cognitive performance later in life [12,13,14]. Third, it is uncertain whether lifestyle and physical development in childhood overcome or diminish the importance of prenatal and neonatal factors in relation to cognitive performance during childhood and adolescence.

The PANIC study (more information about the study in the Methods Section) followed children (aged six to nine years) until adolescence (aged 15 to 17 years) and contains a dataset with extensive information on factors of the child’s and adolescent’s developmental period [15]. Thus, allowing for a comprehensive analysis of the determinants of cognitive performance in children and adolescents. We appraised all the variables collected in the PANIC study and selected the ones that were associated with cognitive performance in previous studies [6,7,8,9,10,11,12,13,14]. Taking advantage of the PANIC study dataset, we evaluated prenatal, neonatal, childhood (at two distinct points) and adolescence factors in relation to cognitive performance during childhood and adolescence.

## 2. Materials and Methods

This is a secondary longitudinal analysis of the PANIC study which is a physical activity and dietary intervention and follow-up study in a population sample of children from eastern Finland. The PANIC study has been described in detail [15]. The study protocol was approved by the Research Ethics Committee of the Hospital District of Northern Savo (Statement 69/2006). The parents or caregivers of the children gave their written informed consent and the children provided their assent to participate. The PANIC study was carried out in accordance with the principles of the Declaration of Helsinki as revised in 2008.

At middle childhood, we assessed 502 children (51.6% girls) of an average age of 7.6 years (range: 6.6 to 9.0 years), at late childhood 437 children (51.0% girls) of 9.8 years of age (range: 8.8 to 11.2 years), and in adolescence 277 participants (54.5% girls) of 15.8 years of age (range: 15.0 to 17.4 years). Table 1 and Table 2 present the descriptive characteristics of the participants.

On average, children were born at 39.8 weeks of gestation (±1.8 weeks). Mothers were 30 years of age on average (range 16 to 44 years), 42.8% did not have any previous births and 31.1% one previous birth; the family income was lower than EUR 30,000 per year for 21.4% of the families and between EUR 30,000 and EUR 60,000 per year for 41.8% of the families.

Children not followed at adolescence presented higher body fat percentage compared to their peers at baseline. A higher proportion of participants born from mothers with preeclampsia were followed at adolescence compared to their peers from mothers who did not have preeclampsia during pregnancy. There was no difference in any other relevant variable because of loss to follow-up.

### 2.1. Patient and Public Involvement

Patients or the public were not involved in the design, or conduct, or reporting or dissemination plans of our research.

### 2.2. Outcome

Cognitive performance was estimated twice in childhood (middle childhood at 6.6 to 9.0 years of age, late childhood at 8.8 to 11.2 years of age) by the Raven’s coloured progressive matrices and by Raven’s progressive matrices in adolescence (15.0 to 17.4 years of age) [16]. Raven’s matrices assess abstract reasoning and fluid intelligence; coloured progressive matrices include 36 and progressive matrices 60 different items [16]. All of the questions on the Raven’s tests consist of visual geometric design with a missing piece. The participant could choose from six to eight choices to pick from to fill in the missing piece. The test becomes increasingly complex, requiring ever-greater cognitive capacity to encode and analyse information for participants as the test progresses [16].

### 2.3. Factors

#### 2.3.1. Socioeconomic Background

Socioeconomic background was assessed by the annual household income (family income) and the level of education of the parents. The family income was asked by a structured questionnaire, at middle childhood, late childhood and adolescence, from both parents and coded into three categories (≤EUR 30,000, EUR 30,001–EUR 60,000 and >EUR 60,000). The level of education was asked by a structured questionnaire from both parents and coded into three categories (vocational school or less, vocational high school, university) based on the highest completed or ongoing degree. If the parents reported different categories, the higher category was used in the analyses. 

#### 2.3.2. Prenatal and Neonatal Exposures

Data were collected on maternal age at child’s birth, gestational age at birth, maternal weight gain, preeclampsia, maternal body mass index (BMI at 1st and 3rd trimesters during pregnancy), gestational diabetes, singleton pregnancy, number of previous births (parity), smoking status during pregnancy (no smoking, smoked but quit during the first trimester, smoked after the first trimester) and also the participants’ birth height, birth weight, independent walking (time in which the child started walking independently) and the one- and five-minute Apgar scores assigned retrospectively from the birth register provided by the National Institute for Health and Welfare. The one- and five-minute Apgar scores evaluate newborns in five criteria: activity (tone), pulse, grimace, appearance and respiration. For each criterion, newborns can receive a score from 0 to 2 [17].

Preeclampsia was defined as hypertension and proteinuria occurring after 20 weeks of gestation. Hypertension was defined as systolic blood pressure ≥140 mmHg or diastolic blood pressure ≥90 mm Hg after 20 weeks of gestation. Proteinuria was defined as the urinary excretion of ≥0.3 g protein in a 24 h specimen, or 0.3 g/L or two ≥1+ readings on dipstick in a random urine determination with no evidence of urinary tract infection.

#### 2.3.3. Pubertal Status

Because only a few children had entered clinical puberty at middle childhood, current height as a percentage of predicted adult height was used as a measure of pubertal status. The boys’ predicted adult height was calculated as follows: the mean of the height of Finnish men (178.6 cm) + [the standard deviation of the height of Finnish men (6.0 cm) × the deviation of the child’s predicted adult height from the average of the predicted adult height of Finnish children]. The girl’s predicted adult height was calculated as follows: the mean of the height of Finnish women (165.3 cm) + [the standard deviation of the height of Finnish women (5.4 cm) × the deviation of the child’s predicted adult height from the average of the predicted adult height of Finnish children]. The deviation in the child’s predicted adult height from the average of the predicted adult height of Finnish children was calculated according to the national guidelines as follows: (the arithmetic mean of the father’s and mother’s height-171)/10 [18].

#### 2.3.4. Child Fitness Exposures

##### Sit and Reach

Lower back and hamstring muscle flexibility were assessed by the sit-and-reach test [19]. The children were asked to sit down with their heels 25 cm apart at the zero line. A measuring stick was placed to −38 cm from the zero line. The children were asked to reach slowly forward as far as possible while keeping the hands parallel and to repeat the same task three times. The test score was the longest distance in cm reached with the fingertips from the starting line of −38 cm, with a smaller distance reached indicating poorer lower back and hamstring flexibility.

##### Handgrip

Handgrip strength was assessed by the Martin vigorimeter (Martin, Tuttlingen, Germany). The children were asked to keep their elbow close to the body, their arm flexed at 90° and to press a rubber bulb maximally three times each with their right and left hand. The mean of the best trial of each hand was used in the analyses and was expressed in kilopascals [19]. 

##### Sit-Up

The sit-up test was used to assess abdominal muscle strength and endurance [19]. The children were asked to lie down with knees flexed at 90°, feet on the ground and arms behind the neck. The children were told to perform as many sit-ups as possible in 30 s with their elbows touching their knees as the assistant held their feet on the floor. The test score was the number of technically correct sit-ups completed in 30 s.

##### Standing Long Jump

Lower limb explosive strength was assessed by the standing long jump test [19]. The children were asked to place their feet next to each other, jump as far as possible and land on both feet. The test score was the best result of three attempts in cm.

##### Shuttle Run

Speed and agility were assessed by the 50 m shuttle run test [20]. Children were asked to run five meters from a starting line to another line as fast as possible, to turn on 25 the line, to run back to the starting line and to repeat until five repetitions were completed. The test score was the running time in seconds, with a longer time indicating a poorer performance.

##### Box and Block

Manual dexterity and upper-limb movement speed were assessed by the box-and-block test [21]. The children were asked to pick up 150 small wooden cubes (2.5 cm/side) one by one with the dominant hand from one side of a wooden box (53.7 cm × 25.4 cm × 8 cm), to move as many cubes as possible to the other side of the box over 60 s and to repeat the same task with the non-dominant hand. The test score was the number of cubes moved to the other side of the box, with smaller number of cubes moved indicating poorer manual dexterity.

#### 2.3.5. Lifestyle Exposures

The following exposures were assessed at middle and late childhood and adolescence. The time the participants’ spent listening to music, playing music, reading, writing, drawing, doing arts and crafts, watching TV and on the computer were inquired by the PANIC Physical Activity Questionnaire administered by the parents with the children [22]. Time spent on each lifestyle factor was queried separately for weekdays and weekends (in minutes per day). The amount of total time for each factor was calculated by adding the times spent in each factor weighted by the number of weekdays and weekend days. We categorised the time in each factor to better represent the time participants spent in each lifestyle factor.

#### 2.3.6. Body Composition and Anthropometrics

Body lean mass and body fat percentage were measured after emptying the bladder, in a supine position and light clothing and after removing all metal objects, by a Lunar^®^ dual-energy X-ray absorptiometry (DXA) device (Lunar Prodigy Advance; GE Medical Systems, Madison, WI, USA) [23]. We excluded the head in the estimation of the participants’ fat percentage. Body weight was measured twice after overnight fasting, after emptying the bladder and standing in light underwear using a calibrated InBody^®^ 720 bioelectrical impedance device (Biospace, Seoul, Korea) to an accuracy of 0.1 kg. The mean of these two values was used in the analyses. Stature was measured three times in the Frankfurt plane without shoes using a wall-mounted stadiometer to accuracy of 0.1 cm. BMI was calculated as body mass (kg) divided by stature (m) squared. The BMI-standard deviation score (BMI-SDS) was calculated based on Finnish references values [24]. The prevalence of underweight, normal weight, overweight and obesity was defined using the national reference values provided by Saari and colleagues [24].

### 2.4. Statistical Analysis

We used STATA version 16 for Windows (StataCorp LP, College Station, TX, USA) and R Studio version 2021.9.0.351 (PBC, Boston, MA, USA) for analysis. Means, standard deviations and absolute and relative frequencies described the variables of interest. Chi-square (categorical variables) or two-way ANOVA (numerical variables) was conducted to evaluate possible differences in the outcome or factors comparing participants with complete data with adolescents not monitored at the second follow-up (dropout analysis). We used hierarchical multilevel linear regressions to evaluate the factors longitudinally associated with cognitive performance. Our hierarchical analysis was conducted in the following steps.

#### 2.4.1. First Step: Defining the Adjustments

In addition to child’s age, sex, pubertal status and intervention group, defined a priori to be included as adjustments, we evaluated which maternal demographic variables (maternal age, family income, gestational age at birth and parity) would be used as adjustments. We ran multilevel linear regressions between each of the abovementioned variables and cognitive performance adjusted for child’s age, sex, pubertal status and intervention group. Maternal age, family income, parity and gestational age at birth were associated with cognitive performance and used as adjustments.

#### 2.4.2. Second Step: Evaluate the Factors Associated with Cognitive Performance

We individually evaluated each of the factors in relation to cognitive performance. All the analyses were adjusted for child’s age, sex, pubertal status, intervention group, maternal age, family income, parity and gestational age at birth. Since we were interested in estimating the longitudinal association between the factors and cognitive performance for each of the life phases (middle childhood, late childhood and adolescence), we included an interaction factor between the factor and time (factor * time).

#### 2.4.3. Third Step: Evaluate the Factors, amongst Domains, Associated with Cognitive Performance

All the factors associated (*p* ≤ 0.05) with cognitive performance in at least one life phase in the second step were included in this third phase, which was conducted in the following order:

1. All the factors with *p* ≤ 0.05 in the prenatal domain plus the variables in the neonatal domain.
2. After selecting all the remaining factors from the prenatal and neonatal domains, we inserted the factors from the child fitness domain into the model.
3. After selecting all the remaining factors from the prenatal, neonatal and child fitness domains, we inserted the factors from the child lifestyle domain in the model.

In this third step, we used the forward stepwise procedure to include and select the factors in the model. We started with the factor with the lowest *p* value. It is important to highlight that we maintained the interaction term between each factor and time in the third step and that we accounted for the cluster structure of data at school level in all multilevel analyses. Preliminary analyses did not observe collinearity between factors in the models. Of note, the associations between factors and cognitive performance were presented as the predicted score in the Raven’s test. Further, to account for the multiple testing, the *p*-values shown are the sharpened False Discovery Rate (FDR) q-values [25].

## 3. Results

**Figure 1:**
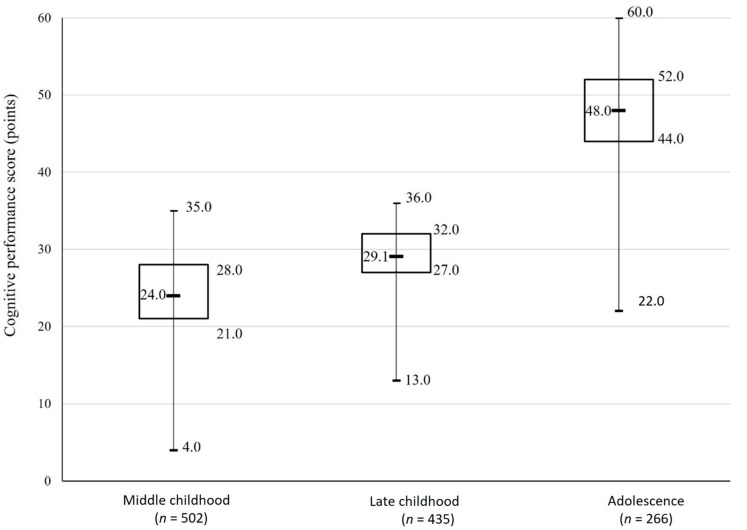
Participants’ cognitive performance during middle childhood, late childhood and adolescence.

Figure 1 presents the cognitive performance of the participants during childhood and adolescence. Children at middle childhood scored on the cognitive performance test, on average, 24.0 points (range 4.0 to 35.0 points), and children at late childhood scored 29.1 points on average (range 13.0 to 36.0 points). Adolescents’ cognitive performance was 48.0 points on average (range 11.0 to 60.0 points).

### 3.1. Prenatal Factors in Relation to Cognitive Performance

From all the prenatal factors tested, only preeclampsia was longitudinally associated with cognitive performance. Particularly, adolescents whose mothers had preeclampsia presented a lower cognitive performance compared to their peers whose mothers did not have preeclampsia (Preeclampsia (No): 50.15, 95% CI 45.44 to 54.86; preeclampsia (Yes): 45.85, 95% CI 39.85 to 51.84; *p* = 0.018; Table 3). However, preeclampsia was not significantly associated with cognitive performance after considering fitness factors (Table 3, Prenatal + Neonatal + Child Fitness model).

### 3.2. Neonatal Factors in Relation to Cognitive Performance

None of the neonatal factors was longitudinally associated with cognitive performance (Table 3).

### 3.3. Child’s Fitness Factors in Relation to Cognitive Performance

From all the child’s fitness factors tested, only the shuttle run and box-and-block tests were longitudinally associated with cognitive performance. One additional second in the shuttle run test was associated with lower cognitive performance at middle childhood (−0.248 points; *p* = 0.038). Each additional score in the box and block test was associated with higher cognitive performance at middle childhood (0.067 points; *p* = 0.001) and adolescence (0.058 points; *p* = 0.010). However, the shuttle run and box and block tests were no longer associated with cognitive performance in the model including Prenatal + Neonatal + Child Fitness factors (Table 3).

### 3.4. Child’s Lifestyle Factors in Relation to Cognitive Performance

A wide range of lifestyle factors was associated with cognitive performance. Specifically, adolescents who listened to music presented lower cognitive performance compared to their peers who did not listen to music. Adolescents who played music showed higher cognitive performance compared to adolescents who did not play music. Reading was longitudinally associated with higher cognitive performance in all developmental phases (middle and late childhood and adolescence). Writing outside the school period was longitudinally associated with lower cognitive performance in middle and late childhood. Adolescents who did arts and crafts showed lower cognitive performance compared to their peers who did not perform arts and crafts. Adolescents who watched TV for more than 30 min/day presented lower cognitive performance compared to their peers who did not watch TV. Children at middle childhood who spent 1 to 60 min/day on the computer exhibited higher cognitive performance compared to their peers who did not dedicate any time to the computer (Table 3, factors individually + adjustments). 

A few lifestyle factors remained associated with cognitive performance in the fully adjusted model (Prenatal + Neonatal + Child Fitness + Child Lifestyle + adjustments model). Particularly, adolescents who listened to music presented lower cognitive performance compared to their peers who did not listen to music. Adolescents who played music exhibited higher cognitive performance compared to their peers who did not play music. Reading was associated with higher cognitive performance in all developmental periods. Writing outside the school period was negatively associated with cognitive performance in all developmental periods (middle and late childhood and adolescence). Adolescents who did arts and crafts showed lower cognitive performance compared to their peers who did not perform arts and crafts. Limited time on the computer (1–60 min/day) was associated with higher cognitive performance at middle childhood and adolescence compared to their peers without time on the computer (Table 3, Prenatal + Neonatal + Child Fitness + Child Lifestyle model).

## 4. Discussion

Several factors were longitudinally associated with cognitive performance in children and adolescents. None of the neonatal and anthropometric parameters was associated with cognitive performance. Preeclampsia (prenatal), listening to music, writing, arts and crafts and watching TV (lifestyle) were negatively associated with cognitive performance. On the other hand, the shuttle run and box and block tests (fitness), and playing music, reading and time on the computer (lifestyle) were positive determinants of cognitive performance in children and adolescents. Fitness and lifestyle factors overcame the importance of prenatal factors on cognitive performance and lifestyle factors were especially relevant in regard to cognitive performance. Of note, reading was the most important positive determinant of cognitive performance during childhood and adolescence.

Our findings highlight the importance of all life periods in relation to cognitive development, from conception to behaviours during adolescence. However, there are some key elements to better understand the importance of each lifetime period in relation to cognitive development. Behaviours incorporated later in life may overcome or diminish the importance of being exposed to previous factors negatively related to cognitive performance.

For example, preeclampsia was negatively associated with cognitive performance in adolescents, as demonstrated previously [26,27]. However, preeclampsia was not associated with cognitive performance after considering the participants’ fitness factors, particularly the shuttle run and the box and block tests. Those are novel and relevant findings because it might be possible to diminish the importance of deleterious factors that the children might have been exposed to during pregnancy or early childhood in relation to cognitive development.

It is complex to interpret the importance of lifestyle factors in relation to cognitive performance. Some lifestyle factors seem to stimulate, whereas others were negatively associated with cognitive performance. Our findings suggest that lifestyle factors play a major role on cognitive performance, particularly during adolescence.

It seems that limited exposure to listening to music, writing, arts and crafts, and watching TV are positively associated with cognitive performance, since dedicating time to those factors was related to worse cognitive performance. Further, listening to music, writing and arts and crafts remained associated with cognitive performance when considering the other lifestyle factors. It is possible that adolescents who spent additional time outside school writing were the ones exhibiting lower cognitive and academic performance at school. Listening to music and arts and crafts during adolescence might be depriving time for reading, playing music and limited computer time (1–60 min/day), which were positively related to cognitive performance. Importantly, future investigations should assess these findings in depth.

It is possible that a wide range of stimuli is beneficial for cognitive performance during childhood and adolescence [28,29,30]. Particularly, playing music during adolescence, 1 to 60 min/day on the computers and reading during childhood and adolescence were related to higher cognitive performance. Further, the more children read, the better their cognitive performance. The importance of reading and reading performance is well established in regard to cognitive function and development in children and adolescents, and recent evidence shows that the relationship might be reciprocal [31]. In summary, our findings support the premise that additional time reading is linked to better cognitive performance during childhood and adolescence.

### Limitations

This study carries limitations that should be considered in the interpretation of the findings. We ran several models, including on the number of factors. Thus, some models might be over fitted. Nevertheless, we also present each factor in relation to cognitive performance adjusted for confounders, similarly to previous studies. We ran various models, but *p*-values were adjusted for multiple testing to diminish the risk of type I errors. This is not a randomised controlled trial; hence, we cannot infer on the causal relationship between factors. We observed differences in body fat percentage and proportion of preeclampsia due to loss to follow-up. Moreover, there might be other factors that might impact cognitive development that we did not account for in our study and should be further investigated, such as genetic [32,33] and nutritional factors [34,35]. 

## 5. Conclusions

Cognitive performance throughout childhood and adolescence was associated with factors from conception to adolescence. Although some prenatal factors were negatively associated with cognitive performance in children, it seems that better fitness and lifestyle factors during childhood can negate these negative relationships. Spending time on a mix of activities seems beneficial and more cognitive challenging lifestyles seem to be more relevant in later childhood and adolescence. Reading is positively associated with cognitive performance, regardless of age, and should be promoted. Practitioners, future interventions and public health policies should be aware of the probable changes in the role of the various lifestyle factors on cognitive performance in different lifetime phases.

## Figures and Tables

**Table 1:** Prenatal and neonatal descriptive characteristics of the participants.

Prenatal	*n*	Mean	SD
Maternal weight gain (kg)	1089	5.2	14.2
BMI (Kg/m²) (1st Trimester)	1203	23.1	4.5
BMI (Kg/m²) (3rd Trimester)	1104	28.3	4.4
**Neonatal**	*n*	Mean	SD
Birth height (cm)	1506	50.0	2.1
Birth weight (g)	1515	3535.1	527.6
Independent walking (months)	912	12.0	1.9
Apgar score 1-min (points)	1277	8.7	0.9
Apgar score 5-min (points)	1277	9.0	0.7
**Prenatal**	*n*	%	
Preeclampsia—No	474	96.3	
Preeclampsia—Yes	18	3.7	
Gestational diabetes mellitus—No	463	92.2	
Gestational diabetes mellitus—Yes	39	7.8	
**Neonatal**	*n*	%	
Small for gestational age—No	371	90.7	
Small for gestational age—Yes	38	9.3	

**Table 2:** Descriptive data on child’s fitness, anthropometrics and lifestyle at middle childhood, late childhood and adolescence.

Variables		Middle Childhood			Late Childhood			Adolescence		
		*n*	Mean	SD	*n*	Mean	SD	*n*	Mean	SD
**Child Fitness**										
Sit and reach (cm)		500	−3.2	8.0	422	−6.0	9.7	249	2.8	12.8
Handgrip strength right (kg)		504	47.8	9.4	435	62.0	13.5	248	106.2	24.7
Handgrip strength left (kg)		504	46.9	9.3	435	61.1	13.6	250	105.6	25.4
Sit up (repetitions)		469	10.6	4.8	398	16.9	4.7	246	21.8	5.8
Standing long jump (cm)		461	125.8	16.5	393	145.3	20.5	242	191.7	31.6
Shuttle run (sec)		456	24.1	2.3	390	22.1	1.7	243	20.6	2.0
Box and block (score)		499	101.7	13.4	433	118.1	12.1	244	139.9	17.2
**Child Anthropometrics**										
BMI (kg/m²)		504	16.1	2.1	437	17.3	2.7	276	21.0	3.6
BMI SDS		504	−0.2	1.1	437	−0.1	1.1	276	−0.1	1.0
Body fat % (excluding the head)		493	21.5	9.1	417	24.6	9.8	265	23.4	10.6
Lean mass % (excluding the head)		493	76.2	8.8	417	72.4	9.7	265	72.9	10.4
		**Middle childhood**				**Late childhood**		**Adolescence**		
			***n***	**%**		***n***	**%**		***n***	**%**
**Child Lifestyle**										
Listening to music	0 min/day		287	57.3		209	28.5		79	28.5
	1–30 min/day		109	21.8		92	21.5		50	18.1
	≥30 min/day		105	21.0		128	29.8		148	53.4
Playing music	Yes		423	84.4		327	76.2		228	82.3
	No		78	15.6		102	23.8		49	17.7
Reading	0 min/day		131	26.2		95	22.1		79	28.5
	1–30 min/day		174	34.7		128	29.8		79	28.5
	≥30 min/day		196	39.1		206	48.0		119	42.9
Writing	0 min/day		336	67.1		322	75.1		103	37.2
	1–14 min/day		67	13.4		50	11.7		32	11.6
	≥15 min/day		98	19.6		57	13.3		142	51.3
Drawing	0 min/day		125	25.0		193	45.0		242	87.4
	>0 min/day		376	75.1		236	55.0		35	12.6
Arts and crafts	0 min/day		268	53.5		304	70.9		244	88.1
	>0 min/day		233	46.5		125	29.1		33	11.9
Watching tv	≤30 min/day		56	11.2		42	9.8		86	31.1
	31–90 min/day		353	70.5		280	65.3		132	47.6
	>90 min/day		92	18.4		107	24.9		59	21.3
Time on the computer	0 min/day		124	24.8		64	14.9		70	25.3
	1–60 min/day		320	63.9		275	64.1		96	34.7
	>60 min/day		57	11.4		90	21.0		111	40.1

**Table 3:** Determinants of cognitive performance in Finnish children at middle childhood, late childhood and adolescence.

Factors	Predicted Cognitive Performance, Scores in the Raven’s Test											
	Factors Individually			Prenatal + Neonatal			Prenatal + Neonatal + Child Fitness			Prenatal + Neonatal+ Child Fitness + Child Lifestyle		
	Raven’s Score	(95% CI)	*p*	Raven’s Score	(95% CI)	*p*	Raven’s Score	(95% CI)	*p*	Raven’s Score	(95% CI)	*p*
**Prenatal**												
Weight gain (kg)—middle childhood	−0.001	(−0.113 to 0.103)	0.926									
Weight gain (kg)—late childhood	0.065	(−0.047 to 0.179)	0.253									
Weight gain (kg)—adolescence	0.078	(−0.062 to 0.217)	0.274									
Preeclampsia												
No—middle childhood	23.21	(20.96 to 25.46)		23.21	(20.96 to 25.46)		23.76	(21.46 to 26.07)				
Yes (ref. (no))—middle childhood	21.09	(17.31 to 24.87)	0.186	21.09	(17.31 to 24.87)	0.053	22.18	(18.32 to 26.05)	0.151			
No—late childhood	29.16	(28.36 to 29.96)		29.16	(28.36 to 29.96)		29.13	(28.36 to 29.90)				
Yes (ref. (no))—late childhood	26.67	(23.51 to 29.84)	0.121	26.67	(23.51 to 29.84)	0.052	26.71	(23.55 to 29.86)	0.105			
No—adolescence	50.15	(45.44 to 54.86)		50.15	(45.44 to 54.86)		49.31	(44.19 to 54.43)				
Yes (ref. (no))—adolescence	45.85	(39.85 to 51.84)	**0.018**	45.85	(39.85 to 51.84)	**0.048**	45.45	(38.87 to 52.03)	0.104			
BMI (kg/m²) (1st trimester)—middle childhood	−0.107	(−0.220 to 0.019)	0.100									
BMI (kg/m²) (1st trimester)—late childhood	−0.060	(−0.187 to 0.067)	0.353									
BMI (kg/m²) (1st trimester)—adolescence	−0.087	(−0.257 to 0.083)	0.315									
BMI (kg/m²) (3rd trimester)—middle childhood	−0.097	(−0.224 to 0.030)	0.134									
BMI (kg/m²) (3rd trimester)—late childhood	−0.033	(−0.167 to 0.101)	0.630									
BMI (kg/m²) (3rd trimester)—adolescence	−0.036	(−0.211 to 0.139)	0.690									
Gestational DM												
No—middle childhood	23.00	(20.71 to 25.29)										
Yes (ref. (no))—middle childhood	24.21	(21.43 to 26.98)	0.193									
No—late childhood	28.98	(28.15 to 29.81)										
Yes (ref. (no.))—late childhood	29.62	(27.71 to 31.53)	0.517									
No—adolescence	49.89	(45.09 to 54.68)										
Yes (ref. (no))—adolescence	50.85	(45.61 to 56.09)	0.460									
**Neonatal**												
Birth height (cm)—middle childhood	0.0.96	(−0.149 to 0.340)	0.443									
Birth height (cm)—late childhood	0.134	(−0.122 to 0.389)	0.306									
Birth height (cm)—adolescence	0.095	(−0.220 to 0.410)	0.554									
Birth weight (kg)—middle childhood	0.001	(−0.001 to 0.001)	0.727									
Birth weight (kg)—late childhood	0.002	(−0.001 to 0.001)	0.654									
Birth weight (kg)—adolescence	0.001	(−0.001 to 0.001)	0.856									
Independent walking (months)—middle childhood	−0.197	(−0.551 to 0.156)	0.274									
Independent walking (months)—late childhood	−0.259	(−0.616 to 0.098)	0.155									
Independent walking (months)—adolescence	−0.299	(−0.711 to 0.113)	0.154									
Apgar 1-min (points)—middle childhood	0.325	(−0.221 to 0.871)	0.244									
Apgar 1-min (points)—late childhood	−0.138	(−0.705 to 0.429)	0.633									
Apgar 1-min (points)—adolescence	0.438	(−0.319 to 1.195)	0.257									
Apgar 5-min (points)—middle childhood	0.303	(−0.409 to 1.016)	0.404									
Apgar 5-min (points)—late childhood	−0.563	(−1.309 to 0.184)	0.140									
Apgar 5-min (points)—adolescence	−0.364	(−1.257 to 0.530)	0.425									
**Child Fitness**												
Sit and reach (cm)—middle childhood	0.009	(−0.059 to 0.077)	0.798									
Sit and reach (cm)—late childhood	0.027	(−0.031 to 0.085)	0.367									
Sit and reach (cm)—adolescence	0.050	(−0.009 to 0.109)	0.094									
Handgrip (right hand) (kg)—middle childhood	−0.050	(−0.108 to 0.008)	0.093									
Handgrip (right hand) (kg)—late childhood	−0.020	(−0.062 to 0.023)	0.363									
Handgrip (right hand) (kg)—adolescence	−0.014	(−0.045 to 0.018)	0.394									
Handgrip (left hand) (kg)—middle childhood	−0.020	(−0.076 to 0.036)	0.486									
Handgrip (left hand) (kg)—late childhood	−0.010	(−0.052 to 0.033)	0.657									
Handgrip (left hand) (kg)—adolescence	−0.001	(−0.032 to 0.030)	0.951									
Sit up (repetitions)—middle childhood	0.003	(−0.112 to 0.118)	0.965									
Sit up (repetitions)—late childhood	−0.025	(−0.158 to 0.108)	0.706									
Sit up (repetitions)—adolescence	0.002	(−0.132 to 0.136)	0.978									
Standing long jump (cm)—middle childhood	−0.004	(−0.038 to 0.030)	0.827									
Standing long jump (cm)—late childhood	−0.006	(−0.036 to 0.024)	0.698									
Standing long jump (cm)—adolescence	−0.011	(−0.035 to 0.014)	0.395									
Shuttle run (sec)—middle childhood	−0.248	(−0.483 to −0.014)	**0.038**				−0.197	(−0.439 to −0.045)	0.104			
Shuttle run (sec)—late childhood	0.099	(−0.258 to 0.455)	0.588				0.103	(−0.254 to 0.460)	0.245			
Shuttle run (sec)—adolescence	−0.368	(−0.746 to 0.010)	0.057				−0.265	(−0.664 to 0.135)	0.110			
Box and Block (score)—middle childhood	0.067	(0.028 to 0.106)	**0.001**				0.039	(−0.006 to 0.093)	0.104			
Box and Block (score)—late childhood	0.011	(−0.035 to 0.057)	0.644				0.107	(−0.040 to 0.061)	0.254			
Box and Block (score)—adolescence	0.058	(0.014 to 0.103)	**0.010**				0.049	(0.001 to 0.096)	0.104			
**Child Lifestyle**												
Listening to music												
0 min/day—middle childhood	23.34	(21.04 to 25.65)								22.96	(20.79 to 25.14)	
1–30 min/day (ref. (0 min/day))—middle childhood	23.40	(20.89 to 25.91)	0.925							23.36	(20.95 to 25.76)	0.307
≥30 min/day (ref. (0 min/day))—middle childhood	22.62	(20.15 to 25.91)	0.272							21.94	(19.58 to 24.29)	0.097
0 min/day—late childhood	29.15	(28.16 to 30.14)								28.79	(27.85 to 29.73)	
1–30 min/day (ref. (0 min/day))—late childhood	29.51	(28.19 to 30.82)	0.612							28.99	(27.72 to 30.27)	0.428
≥30 min/day (ref. (0 min/day))—late childhood	28.64	(27.49 to 29.79)	0.430							28.32	(27.23 to 29.41)	0.296
0 min/day—adolescence	51.04	(46.13 to 55.95)								52.37	(47.66 to 57.07)	
1–30 min/day (ref. (0 min/day))—adolescence	49.67	(44.67 to 54.66)	0.192							49.71	(44.97 to 54.46)	**0.041**
≥30 min/day (ref. (0 min/day))—adolescence	49.18	(44.36 to 54.00)	**0.025**							49.92	(45.35 to 54.48)	**0.040**
Playing music												
No—middle childhood	22.94	(20.65 to 25.25)								22.72	(20.55 to 24.90)	
Yes (ref. (no))—middle childhood	22.91	(20.39 to 25.42)	0.949							22.70	(20.31 to 25.08)	0.458
No—late childhood	29.10	(28.23 to 29.97)								28.70	(27.87 to 29.53)	
Yes (ref. (no))—late childhood	28.79	(27.53 to 30.04)	0.630							28.63	(27.43 to 29.83)	0.458
No—adolescence	49.87	(45.10 to 54.63)								50.51	(45.96 to 55.05)	
Yes (ref. (no))—adolescence	52.46	(47.35 to 57.56)	**0.006**							53.16	(48.24 to 58.07)	**0.040**
Reading												
0 min/day—middle childhood	22.12	(19.71 to 24.53)								21.71	(19.36 to 24.06)	
1–29 min/day (ref. (0 min/day))—middle childhood	22.07	(19.74 to 24.40)	0.934							21.79	(19.52 to 24.05)	0.458
≥30 min/day (ref. (0 min/day))—middle childhood	24.39	(22.08 to 26.69)	**<0.001**							24.35	(22.15 to 26.54)	**0.031**
0 min/day—late childhood	27.56	(26.28 to 28.83)								27.03	(25.74 to 28.33)	
1–29 min/day (ref. (0 min/day))—late childhood	29.09	(27.97 to 30.21)	**0.042**							28.86	(27.75 to 29.98)	**0.041**
≥30 min/day (ref. (0 min/day))—late childhood	29.61	(28.65 to 30.58)	**0.003**							29.53	(28.61 to 30.44)	**0.030**
0 min/day—adolescence	48.89	(44.09 to 53.68)								48.60	(43.88 to 53.32)	
1–29 min/day (ref. (0 min/day))—adolescence	51.13	(46.33 to 55.93)	**0.015**							51.84	(47.16 to 56.52)	**0.041**
≥30 min/day (ref. (0 min/day))—adolescence	50.53	(45.76 to 55.31)	0.051							51.82	(47.13 to 56.50)	**0.041**
Writing												
0 min/day—middle childhood	23.58	(21.27 to 25.89)								23.21	(21.01 to 25.41)	
1–14 min/day (ref. (0 min/day))—middle childhood	21.83	(19.30 to 24.35)	**0.018**							21.20	(18.79 to 23.62)	**0.041**
≥15 min/day (ref. (0 min/day))—middle childhood	23.75	(21.27 to 26.22)	0.798							22.33	(19.93 to 24.73)	0.155
0 min/day—late childhood	29.37	(28.48 to 30.26)								29.40	(28.57 to 30.24)	
1–14 min/day (ref. (0 min/day))—late childhood	29.18	(27.56 to 30.81)	0.822							28.63	(27.05 to 30.22)	0.252
≥15 min/day (ref. (0 min/day))—late childhood	27.73	(26.21 to 29.24)	**0.035**							27.01	(25.51 to 28.52)	**0.041**
0 min/day—adolescence	49.82	(45.00 to 54.64)								51.80	(47.11 to 56.49)	
1–14 min/day (ref. (0 min/day))—adolescence	51.95	(46.82 to 57.08)	0.076							51.83	(46.89 to 56.76)	0.458
≥15 min/day (Ref (0 min/day))—adolescence	48.66	(43.84 to 53.48)	0.133							48.76	(44.16 to 53.36)	**0.041**
Drawing												
0 min/day—middle childhood	22.90	(20.45 to 25.35)										
≥0 min/day (ref. (0 min/day))—middle childhood	23.32	(21.02 to 25.63)	0.494									
0 min/day—late childhood	28.80	(27.78 to 29.83)										
≥0 min/day (ref. (0 min/day))—late childhood	29.30	(28.33 to 30.28)	0.387									
0 min/day—adolescence	49.47	(44.67 to 54.28)										
≥0 min/day (ref. (0 min/day))—adolescence	51.33	(46.22 to 56.43)	0.096									
Arts craft												
0 min/day—middle childhood	22.36	(20.02 to 24.70)								22.38	(20.16 to 24.60)	
≥0 min/day (ref. (0 min/day))—middle childhood	23.39	(21.06 to 25.71)	0.055							23.39	(21.18 to 25.59)	0.059
0 min/day—late childhood	28.96	(28.07 to 29.85)								28.54	(27.67 to 29.42)	
≥0 min/day (ref. (0 min/day))—late childhood	29.10	(27.94 to 30.25)	0.827							28.97	(27.86 to 30.07)	0.296
0 min/day—adolescence	50.83	(46.04 to 55.63)								52.29	(47.68 to 56.89)	
≥0 min/day (ref. (0 min/day))—adolescence	47.80	(42.80 to 52.79)	**0.005**							48.53	(43.76 to 53.30)	**0.041**
Watching tv												
≤30 min/day—middle childhood	21.94	(19.17 to 24.72)								21.79	(19.15 to 24.44)	
31–90 min/day (ref. (≤30 min/day))—middle childhood	23.46	(21.16 to 25.75)	0.097							22.99	(20.81 to 25.18)	0.133
>90 min/day (ref. (≤ 30 min/day))—middle childhood	22.73	(20.26 to 25.20)	0.453							22.50	(20.15 to 24.85)	0.296
≤30 min/day—late childhood	30.34	(28.43 to 32.25)								30.04	(28.21 to 31.87)	
31–90 min/day (ref. (≤ 30 min/day))—late childhood	28.68	(27.77 to 29.58)	0.090							28.24	(27.39 to 29.09)	0.059
>90 min/day (ref. (≤30 min/day))—late childhood	29.53	(28.34 to 30.73)	0.447							29.13	(27.28 to 30.28)	0.246
≤30 min/day—adolescence	51.06	(46.23 to 55.89)								52.35	(47.75 to 56.95)	
31–90 min/day (ref. (≤30 min/day))—adolescence	49.08	(44.25 to 53.92)	**0.013**							50.74	(46.12 to 55.36)	0.050
>90 min/day (ref. (≤30 min/day))—adolescence	49.00	(44.09 to 53.91)	**0.035**							51.0	(46.27 to 55.73)	0.133
Time on the computer												
0 min/day—middle childhood	22.14	(19.68 to 24.60)								21.83	(19.49 to 24.17)	
1–60 min/day (ref. (0 min/day))—middle childhood	23.68	(21.38 to 25.97)	**0.015**							23.14	(20.95 to 25.32)	**0.044**
>60 min/day (ref. (0 min/day))—middle childhood	22.87	(20.20 to 25.55)	0.448							22.41	(19.85 to 24.97)	0.307
0 min/day (ref. (0 min/day))—late childhood	28.99	(27.43 to 30.56)								28.85	(27.36 to 30.34)	
1–60 min/day (ref. (0 min/day))—late childhood	29.30	(28.41 to 30.20)	0.705							28.80	(27.93 to 29.67)	0.458
>60 min/day (ref. (0 min/day))—late childhood	28.33	(27.02 to 29.63)	0.487							28.19	(26.94 to 29.45)	0.296
0 min/day—adolescence	49.46	(44.51 to 54.31)								49.73	(45.07 to 54.38)	
1–60 min/day (ref. (0 min/day))—adolescence	50.56	(45.72 to 55.40)	0.234							51.85	(47.21 to 56.50)	**0.042**
>60 min/day (ref. (0 min/day))—adolescence	48.97	(44.09 to 53.84)	0.596							49.95	(45.27 to 54.63)	0.434
**Child Anthropometrics**												
BMI (kg/m²)—middle childhood	−0.017	(−0.269 to 0.235)	0.893									
BMI (kg/m²)—late childhood	0.075	(−0.136 to 0.285)	0.488									
BMI (kg/m²)—adolescence	0.800	(−0.150 to 0.310)	0.495									
BMI SDS—middle childhood	0.066	(−0.415 to 0.548)	0.787									
BMI SDS—late childhood	0.111	(−0.413 to 0.634)	0.678									
BMI SDS—adolescence	0.184	(−0.551 to 0.919)	0.624									
Body fat % (excluding the head)—middle childhood	0.009	(−0.050 to 0.068)	0.758									
Body fat % (excluding the head)—late childhood	0.019	(−0.041 to 0.079)	0.543									
Body fat % (excluding the head)—adolescence	0.044	(−0.026 to 0.115)	0.221									
Lean mass % (excluding the head)—middle childhood	−0.009	(−0.070 to 0.053)	0.777									
Lean mass % (excluding the head)—late childhood	−0.018	(−0.079 to 0.043)	0.565									
Lean mass % (excluding the head)—adolescence	−0.043	(−0.115 to 0.029)	0.238									

Legend: Results are presented in the predicted values of the Raven’s score. All analyses were adjusted for child’s age, sex, pubertal status, intervention group, and maternal age, family income, parity and gestational age at birth. *p*-values are the sharpened False Discovery Rate q-values.

## Data Availability

The data is not publicly available due to ethical reasons. However, Timo A. Lakka can provide further information on the PANIC study and the PANIC data on a reasonable request.
